# Neonatal palliative care: Assessing the nurses educational needs for terminally ill patients

**DOI:** 10.1371/journal.pone.0280081

**Published:** 2023-01-06

**Authors:** Omar M. Khraisat, Ahmad M. Al-Bashaireh, Raed Khafajeh, Ola Alqudah

**Affiliations:** 1 Faculty of Nursing, Al-Ahliyya Amman University, Amman, Jordan; 2 Health Sciences Division-Nursing, Higher Colleges of Technology, Fujairah, United Arab Emirates; 3 Nursing Education Department, King Fahad Medical City, Riyadh, KSA; 4 Consultant-Family Medicine, King Fahad Medical City, Riyadh, KSA; Waikato Institute of Technology, NEW ZEALAND

## Abstract

**Background:**

For terminally sick neonates and their families, it’s crucial to provide holistic nursing care that incorporates both curative and palliative care as much as feasible. It is well known that the biggest obstacle to delivering palliative care for neonatal children is a lack of training for nurses. Aim: The aim of this research is to investigate the experiences of nurses who provide care for neonates who are terminally ill as well as their educational requirements for neonatal palliative care.

**Method:**

A cross-sectional descriptive study was conducted among 200 nurses working in a tertiary center providing care for terminally ill neonates in Saudi Arabia. Data was collected from using Neonatal Palliative Care Questionnaire (QNPC) from January 2021 to March 2021.

**Results:**

Two hundred nurses were surveyed (the response rate was 79%). The mean age of the 158 participants was 35.67 (standard deviation (SD): 7.43), and the majority were female (151; 95.6%). The majority were bachelor’s holders (119; 75.3%), with more than 5 years of experience in providing care for neonates (100; 63.3%). Most of the participants reported not receiving any education about palliative care (115; 72.8%). Nurses reported a moderate level of experience in all areas of neonatal palliative care. The total mean score of palliative care experiences of neonates was 3.42 (SD: 1.35). However, the majority of nurses reported little experience discussing the transition period to palliative care for neonates 2.95 (SD: 1.93), the discussion of code status (DNR) during terminal illness of neonates 3.11 (SD: 1.54) and spiritual support 2.90 (SD: 1.55).

**Conclusion:**

The assessment of the fundamental skills of neonatal palliative care by nurses was insufficient. To enhance the quality of care, it is crucial to incorporate education on neonatal palliative care into programs for nursing staff development.

## Introduction

The late twentieth century brought about a significant change in neonatal care. Many hospitals consistently save and treat critically extremely sick newborns that are born within 22 to 24 weeks. However, due to prematurity and multifaceted medical problems, newborns still die [1,2]. In Saudi Arabia, neonatal palliative care is a practical option [3,4]. There has been a rise in interest in palliative care integration across the globe. The World Health Organization announced the necessity of palliative care education and training for healthcare professionals as part of a public health strategy [4,5]. The greatest obstacle to delivering palliative care for neonatal infants is inadequate training of nurses, as is well-documented [1,2]. Nurses who provide palliative care to these newborns must possess specialized skills to do so efficiently [5,6].

It focuses on neonates and their families. It may primarily be integrated with cure, the progress of disease care, and then exaggerated when that form of care no longer achieves the cure-oriented goal [7–10].

Many studies revealed uncertainty, discomfort, and chaos among nurses about the prognosis of the disease, the management and treatment of symptoms, and the appropriate time to transition from curative to palliative care [11–15]. These studies revealed that education works as the foundation for competency development that focuses on delivering the best care possible to neonates who are dying and their families [12–14].

Numerous studies highlighted the need to enhance nurses’ confidence in palliative care and grief through continuing education [6,14,16]. Likewise, to relieve anxiety among healthcare providers, continuing educational training is crucial when providing neonatal palliative care, namely when communicating DNR options with families and pain management for dying neonates [12,13,17,18].

Saudi Arabia is unique compared to western nations where the idea of palliative care is first introduced. Two decades ago, hospice and palliative care were provided to patients in Saudi Arabia who were near death. Doctor Isbister founded this service at the King Faisal Specialist Hospital and Research Center (KFSH&RC) in Riyadh, and it has quietly evolved since then [19].

The philosophy of palliative care should be integrated within health care covering all stages of illness [20,21]. However, the education of palliative care for nurses is crucial [3–5,20,22].

In Saudi Arabia, little is identified about neonates’ palliative care among nurses. Overall, the necessity for palliative care has increased significantly. There is a real need for palliative care in Saudi Arabia for neonates [5,6]. Similar to that, this study takes into account a baseline evaluation to provide standardized nursing education for caring for neonates who are nearing the end of their lives, and there has even been limited research on this topic. This study aimed to evaluate the experiences of neonatal palliative care among nurses and their educational needs. The research question was related to what are the current palliative care experiences provided by nurses for terminally ill neonates and their educational needs.

## Methods

### Study design and sampling

A non-experimental, cross-sectional, descriptive design was used. The study was carried out in a Specialized Tertiary Hospital for Children and Women located in Saudi Arabia. Nurses who provided care for newborns and their families who could read and understand written English were required for inclusion in this study.

A convenience sample was utilized. A sample size was determined based on Cohen’s (1992) guidelines using a power = 0.80, alpha (α) = 0.05, and medium effect size = 0.25 two hundred participants were required [23].

### Instrument

The study utilized anonymous self-reported questionnaires. The questionnaire is composed of two parts. The first part of the questionnaire includes demographic characteristics, including age, gender, education level, and whether or not they had undergone a neonatal palliative educational program. The second part includes the English version of the Neonatal Palliative Care Questionnaire (QNPC), developed by Peng et al. (2013), was used to measure nurses’ experiences of palliative care among neonates. QNPC comprises eight items; the responses being never (1), rarely (2), sometimes (3), often (4) or, always (5) [2]. The score ranges from 8 (minimum score) to 40 (maximum score). The sum of the scores for each QNPC item divided by the number of items was calculated to obtain the mean QNPC score. The mean score was used as a cutoff point to measure the similar to the experience and knowledge on neonatal palliative care [2]. Therefore, the higher one scores above the mean, the higher the experience and knowledge level. The content validity of the questionnaire was reviewed by two of the academic staff with PhD degrees in pediatric health nursing.

### Pilot study

A pilot study was conducted to test the instrument’s psychometric properties and the time required to complete the questionnaire and clarity. Twenty nurses completed the questionnaire within 5–15 minutes. The QNPC reliability revealed an alpha coefficient of 0.90. However, Peng et al. reported that the QNPC Cronbach alpha coefficient was 0.94 [2].

### Data collection

Approval to conduct the study was obtained from the Institutional Review Board (IRB) at the King Fahad Medical City, Riyadh-Kingdom of Saudi Arabia. Data was collected from January 2021 to March 2021. The nurses were approached in the private room setting. The questionnaire was distributed at the end of the shift. Participants were informed about the purposes of the study. They were provided with the questionnaire along with a cover letter.

### Ethical considerations

King Fahad Medical City in Riyadh, Kingdom of Saudi Arabia, provided institutional review board approval (IRB00010471). Institutional review board approval was obtained from King Fahad Medical City, Riyadh-Kingdom of Saudi Arabia (IRB00010471). The participants had full disclosure about the risks and benefits of the study. They were assured there was no risk. They were also assured that participation was voluntary and they could withdraw from the study at any time without any. In addition, they were assured that all the information obtained would be anonymous by assigning numbers to participant’s questionnaire, keeping it in locked place and deleting the data completely once the study was concluded.

### Data analysis

Data were analyzed using SPSS version 22 for Windows (SPSS Inc., Chicago, Illinois). Mistake entry, missing data, and outliers were screened. The data was numerically coded and statistically analyzed in a descriptive manner. Descriptive statistics (frequency, mean, and standard deviation) were used to analyze the findings (SD).

## Results

The targeted sample comprises (sample size [N]: 200) nurses, the response rate was (158; 79%). The mean age was 35.67 years (SD: 7.43). The majority of the participants were female (151; 95.6%), with Bachelor’s holders (119; 75.3%), and had more than 5 years of experience in providing care for neonates (100; 63.3%). The majority of the participants held the staff nurse position (98; 62%). Most of the participants reported not receiving any education about palliative care (115; 72.8%). Furthermore, a high percentage of junior nurses (less than 2 years of professional experience) and nurses awarded with a diploma degree reported inadequate training in neonatal palliative care (68.2% and 83.3%, respectively) (Table 1).

**Table 1:** Demographic characteristics of nurses working in Saudi Arabia at Children and Women Hospital (N = 158).

	*n*	%
Age Mean (SD)	35.67	7.43
Gender		
Male	7	4.4
Female	151	95.6
Educational degree		
Diploma degree	30	19
Bachelor’s degree	119	75.3
Higher degree	9	5.7
Professional experience in neonatal care		
< 2 years	22	13.9
2–5 years	36	22.8
> 5 years old	100	63.3
Professional title		
Charge nurse	14	8.9
Staff Nurse I	98	62
Staff Nurse II Nationality Saudi Non-Saudi Receive education in neonatal palliative care Yes No	46 16 142 43 115	29.1 10.1 89.9 27.2 72.8
Receive education in neonatal palliative care according to the professional experience		
Professional experience < 2 years		
Yes	7	31.8
No	15	68.2
Professional experience 2–5 years		
Yes	8	22.2
No	28	77.8
Professional experience > 5 years		
Yes	26	26
No	74	74
Receive education in neonatal palliative care according to the educational degree		
Diploma degree		
Yes	5	16.7
No	25	83.3
Bachelor’s degree		
Yes	33	27.7
No	86	72.3
Higher degree		
Yes	3	33.3
No	6	66.7

### Experience in providing palliative care for neonates

The total mean score of palliative care experiences of neonates was 3.42 (SD: 1.35; range: 8–40). Nurses reported that they were routinely visible to death in the neonatal intensive care unit (NICU) with a mean score of 4.20 (SD: 1.26). Moderate experiences were reported in pain management 3.43 (SD: 1.33), recognizing signs of imminent death 3.60 (SD: 1.18), and symptomatic care 4.17 (SD: 1.14). However, the mean scores of the item belonging to the experience of discussion of code status (DNR) during terminal illness of neonates, the family conference to discuss a newly diagnosed life-threatening illness, discussion of the transition period to palliative care for neonates, and spiritual care were low (mean: 3.11, 2.97, 2.95, 2.90, respectively) (Table 2).

**Table 2:** Experiences of nurses with neonatal palliative care for terminally ill patients (N = 156).

Items	Mean	SD
I am exposed to death in the NICU Providing symptomatic care for terminally ill neonates (i.e., skincare, comfort positioning).	4.20 4.17	1.26 1.14
Recognizing the signs of impending death in terminally ill neonates.	3.60	1.18
Providing adequate pain control for terminally ill neonates.	3.43	1.33
Discussing code status with families of terminally ill neonates.	3.11	1.54
Conducting a family conference to discuss a newly diagnosed life-threatening illness in neonates.	2.97	1.41
Discussing with parents the transition from a curative approach to a palliative approach in caring for the terminally ill neonate.	2.95	1.39
Providing spiritual support for families with dying neonates.	2.90	1.55

The most important pre-educational topics in neonatal palliative care, according to nurses, were starting coordinating the care of dying neonates, how to conduct a family conference to discuss a recently diagnosed life-threatening illness, the transition from curative to palliative care, discussing DNR with families, and promoting the confidence of junior nurses by serving as a mentor or role model to younger colleagues. The scale for evaluating pre-educational topics ranged from 1 to 12.Around mean scores of 2.33 (SD: 3.00) and 6.44 (SD: 3.98). However, the least important subjects were pain and symptom treatment. The findings indicated that Saudi Arabian nurses lack proper training in neonatal palliative care.

## Discussion

It can be distressing for nurses to provide end-of-life care to pediatric patients and their families since they may not have the necessary training to handle the situation [6]. The confusion around death might be upsetting for nurses who have chosen to deal with neonates. According to the study’s findings, nurses lack sufficient experience with the fundamentals of providing palliative care for neonates. The development of interventions to deliver high-quality end-of-life care for newborns might result from a better understanding of the educational needs for palliative care that nurses need. The study’s findings revealed that the QNPC mean score was on average quite low the reported nurses’ level of experience in providing neonates end-of-life care is generally low, from coordinating care of end-of-life for neonate’s patients to providing spiritual to family. These findings suggested that end-of-life teaching for neonates may predominantly take place informally (i.e., through personal and clinical experiences). Additionally, knowing how to provide for a neonate’s end-of-life care frequently relies heavily on trial and error [1,2]. Furthermore, practical training is insufficient and ineffective in improving their ability to provide end-of-life care for newborns while at work [2,5,6].

Together, these results offer compelling evidence that formalized training in neonate end-of-life care is necessary; earlier studies indicated lower means [2,17].

This study is consistent with Chin (2020) and Peng et al. (2013) studies in which the nurses reported lacking experience with neonates end-of-life care, specifically in the management of symptoms. They also reported feeling particularly distressed when a neonates patient appeared to be in pain and the family needed spiritual support [2,17]. Nurses who lack experience providing end-of-life care may feel less competent and are more likely to experience burnout, inadequacy, and discomfort [2,6]. Therefore, the majority of nurses receive inadequate formal training in end-of-life care, despite the existence of standards regarding the requirement for instruction in caring for terminally ill neonatal patients throughout training [2,4,6,16].

This situation might be connected to how tense and stressful it is for parents and other family members when a loved one passes away. Additionally, parents claimed that their stress levels are rising as a result of watching their child suffer and worrying about the child’s death, particularly because they are unable to protect their child and are unaware of the [5,8,13,15]. Parents who experience these stressors often feel very helpless, and they require empathetic assistance from caregivers; nurses are well-positioned to offer this support [5,8,13,15]. As a result, nurses must set aside time to listen when family members need to express their emotions, offer consoling words, and have an honest conversation about grieving.

The results of this study were partly consistent with earlier research on participants with little experience [2,10]. However, there were observable variations that might be connected to Saudi Arabia’s palliative care system. In comparison to comparable studies from other regions of the world [2,10], the mean score for nurses’ experience in this study was greater. Additionally, nurses lacked a basic awareness of the fundamentals of palliative care, such as addressing DNR with families and comprehending the transition from curative to palliative care. This is because Saudi Arabia has no formal curriculum for nursing professionals in neonatal palliative care [3,5]. Ahead, the limitation of palliative care services and education programs [3,5]. The fact that most nurses are foreigners who immigrated to Saudi Arabia with varied backgrounds and cultural influences also explains this. They require further training in Islamic culture and how to provide spiritual care to a kid who is terminally sick [20,21].

In addition, nurses reported a poor experience in holding a family conference to discuss a newly diagnosed life-threatening disease in neonates. In order to support the transition stage of palliative care for newborns, it may be necessary to train nurses in the abilities of team leader, facilitator, news breakdown, and coordination with other specialties such as social workers, psychologists, psychiatrists, and palliative care specialists [3,5,6]. Additionally, nurses reported a poor experience in providing spiritual support to families with dying neonates. According to Khraisat et al. (2019, page 5) [21], spirituality in Islam is defined by "how individuals pursue and communicate meaning and purpose through their relationship with Allah" [21]. It has long been recognized that spirituality is a crucial component of patient care. According to the study by Khraisat et al. (2019), nurses need to be educated and trained in order to give spiritual care. To ascertain the competence, distribution, accessibility, and necessity of neonatal palliative care in Saudi Arabia, more research is required.

The low mean score of experience in providing this care may be due to the paucity of educational courses on palliative care for newborns offered to nurses for staff development. Numerous studies conducted in Saudi Arabia urged nursing schools to include palliative care in their curricula [4,6]. The need to incorporate neonatal palliative care into hospital staff development programs and guidelines, however, requires additional attention. Even though all words are thought to be open to misinterpretation by Diploma holders, using the English version of the questionnaire may make it more difficult for nurses to understand specific themes.

The findings show that neonatal palliative care services are essentially nonexistent as a result of a lack of expertise and training in this field. Additionally, nurses reported the significance and necessity of core competencies in neonatal palliative care, such as talking about code status (DNR) during terminal illness in neonates, holding a family conference to talk about a recently discovered life-threatening illness, talking about the transition period to palliative care for neonates, and providing spiritual care in Islamic culture.

## Limitations

The study does have certain limitations. First off, convenience sampling was used to choose the sample, thus the experiences of participants who took the survey may not entirely mirror those of those who did not. As a result, it is less likely that the study’s implications will be broadly applied. Second, the external validity of the results is constrained by the fact that this study was restricted to a single hospital located in Riyadh, the capital of Saudi Arabia. Third, using a self-report questionnaire may create bias since participants might not always provide accurate accounts of their neonatal palliative experience.

## Conclusion

The main aim of this study was to assess the experiences of nurses in palliative care for neonates According to the results presented; the experience of nurses was inadequate in the core competencies of palliative care for the neonates, as shown by the low mean QNPC score.

Additionally, fewer nurses than those who did not undergo palliative education for neonates. However, the lack of education courses on palliative care for neonates in nursing development programs in the context of Arabic Islamic culture may be the cause of the nurses’ lack of expertise with this type of care. Furthermore, there is no hospital-based program for neonatal palliative care. Numerous experts have criticized the palliative care nursing training hours as being insufficient [4,6,22]. Furthermore, according to their educational backgrounds, nurses who did not obtain neonatal palliative care training had diploma, bachelors, and higher degree holders (respectively, 83.3%, 72.3%, and 66.7%). The importance of including the subject of neonatal palliative care in nursing schools and hospital education programs is highlighted by this finding. Therefore, nursing curriculum should include the fundamental competencies of palliative care for neonates in order to strengthen nurses’ competencies in this area. These skills could include dealing with pain, managing symptoms, navigating the transition from palliative care to spirituality, and providing spiritual support. Additionally, numerous researches [5,22] have stressed how critical it is to advance nursing practice by incorporating palliative care into the curriculum.

Consequently, the quality of care will be impacted by nurses’ insufficient knowledge of neonatal palliative care. The mandatory inclusion of neonatal palliative care instruction in nursing curricula is advised. To evaluate the availability, capability, and distribution of palliative care for neonatal patients in Saudi Arabia, more research is advised namely a qualitative.

## Supporting information

S1 Dataset (XLSX)

S1 File (ZIP)
