# Supplementary figures and images for: Neonatal palliative care: Assessing the nurses educational needs for terminally ill patients
Source: PLoS One. 2023 Jan 6;18(1):e0280081. doi: 10.1371/journal.pone.0280081 (PMC9821778; doi:10.1371/journal.pone.0280081)

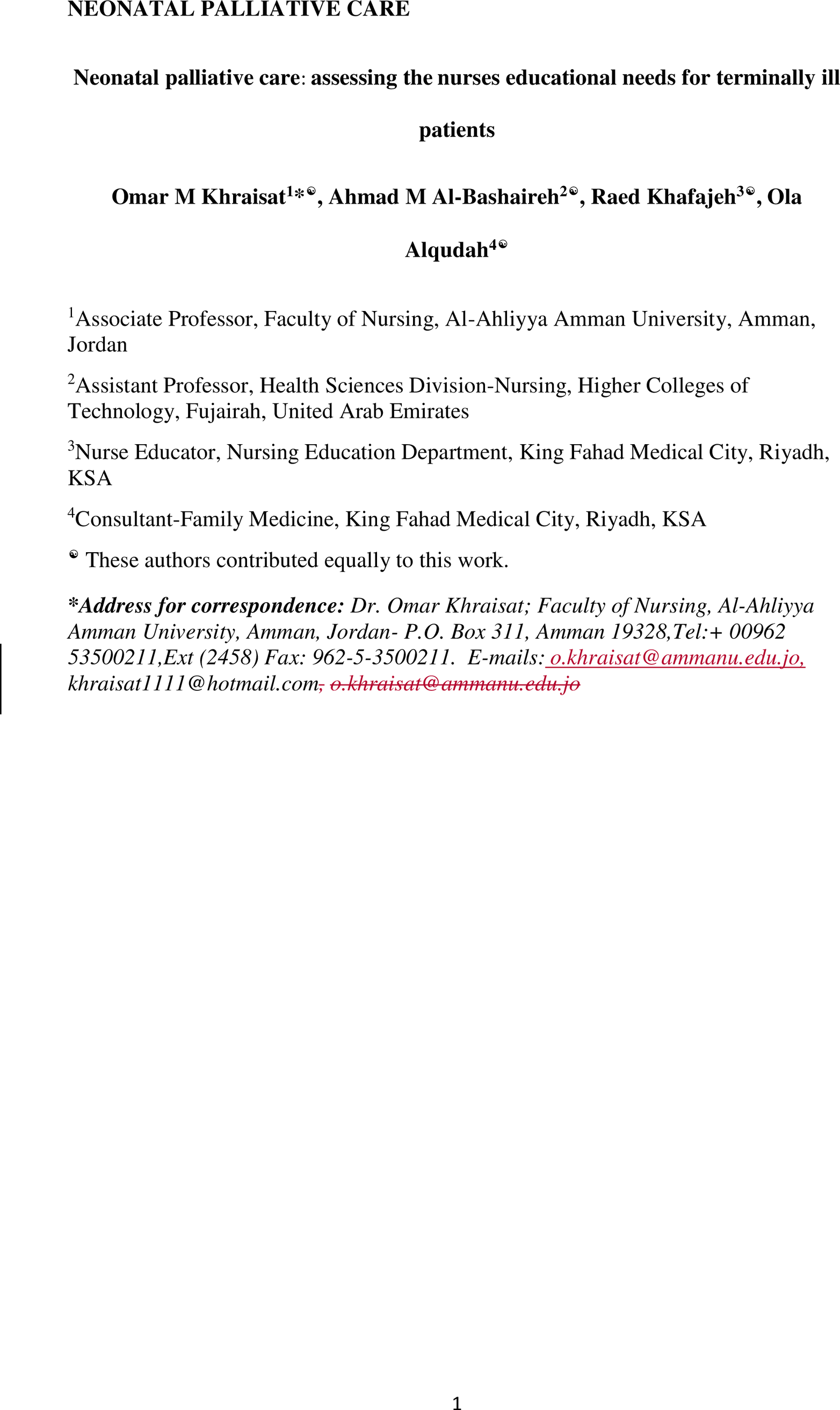

Supplement: S1 File — (ZIP) [file pone.0280081.s002.zip › PACE Corrected/locked tracked changes.tif]

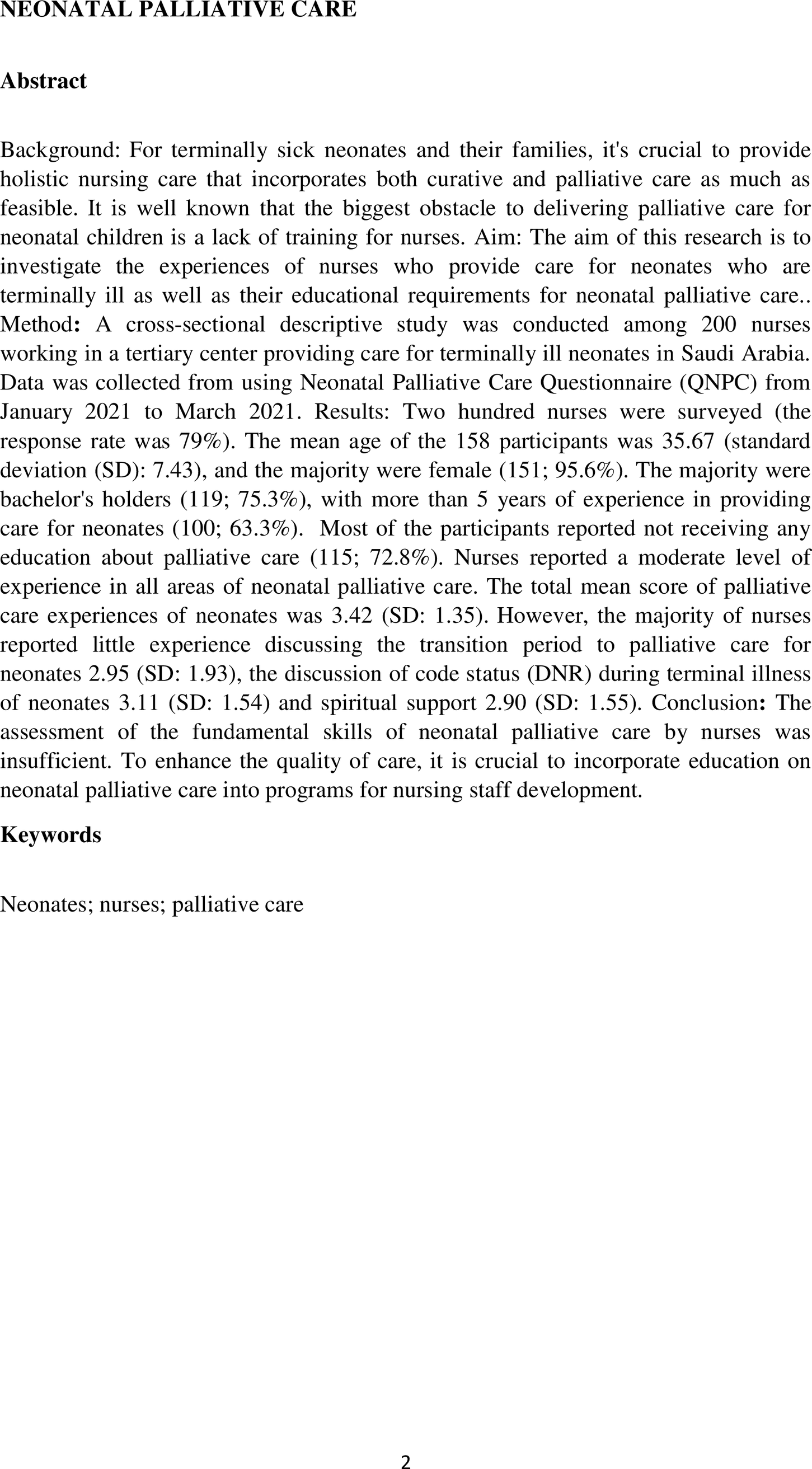

Supplement: S1 File — (ZIP) [file pone.0280081.s002.zip › PACE Corrected/locked tracked changes.tif]

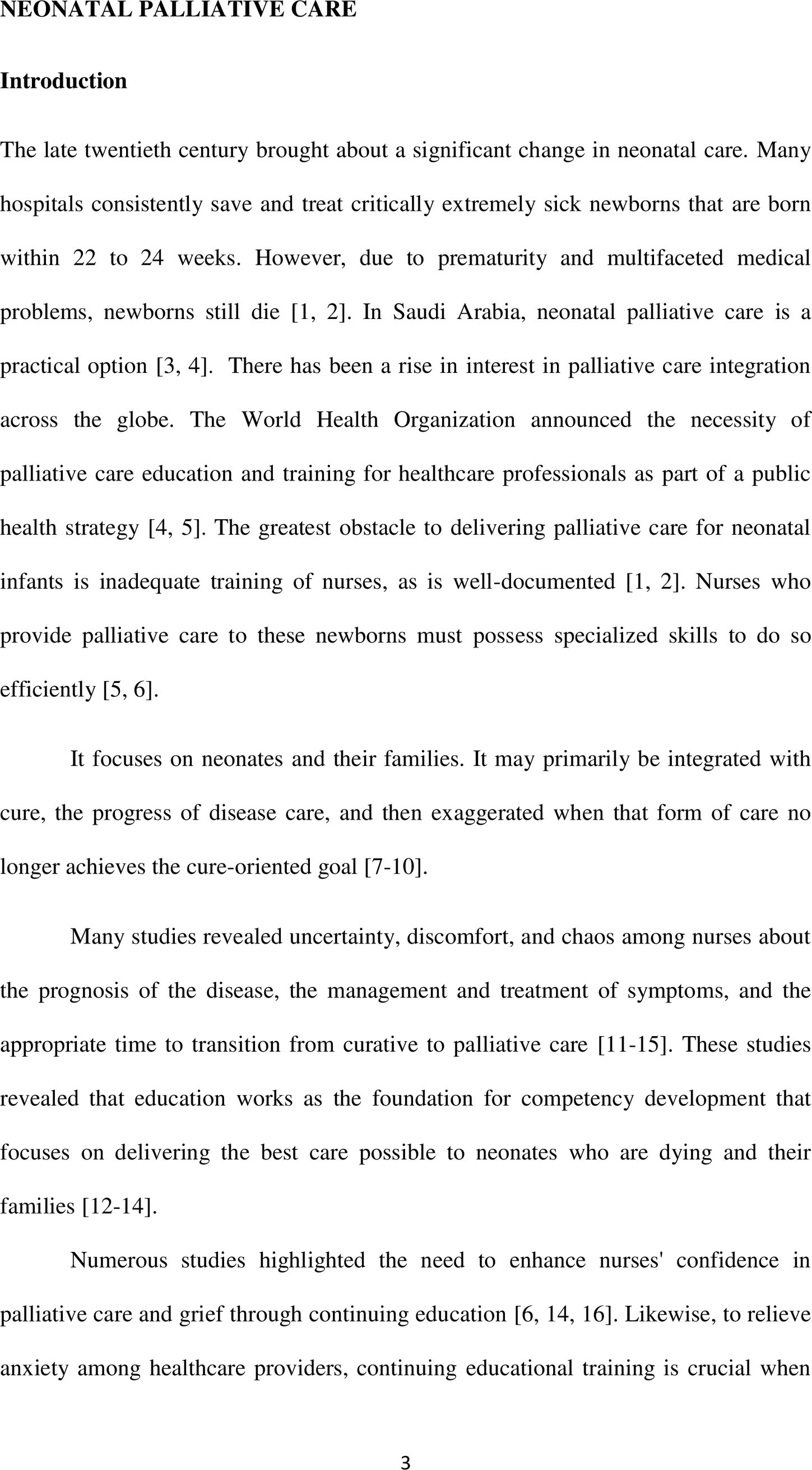

Supplement: S1 File — (ZIP) [file pone.0280081.s002.zip › PACE Corrected/locked tracked changes.tif]

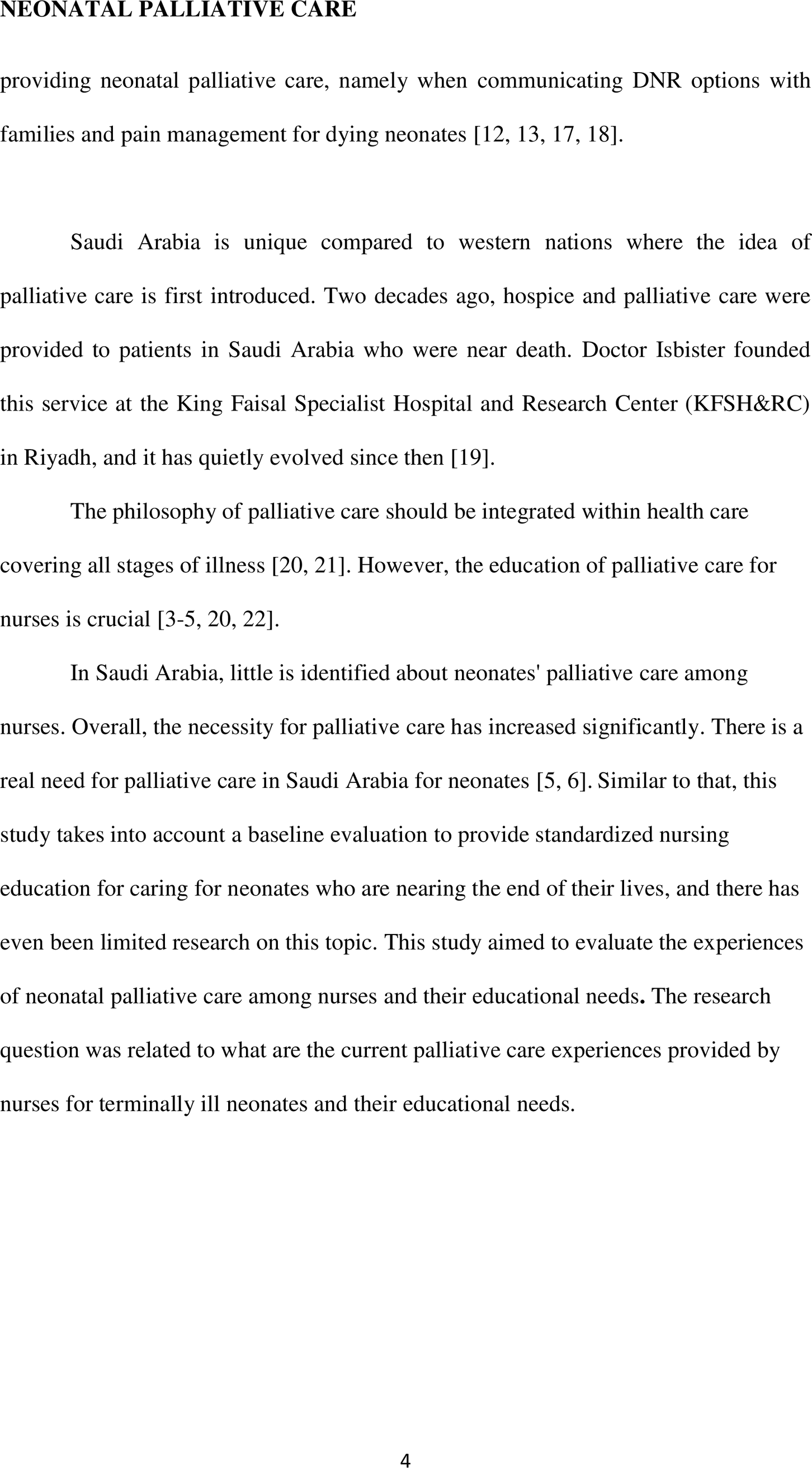

Supplement: S1 File — (ZIP) [file pone.0280081.s002.zip › PACE Corrected/locked tracked changes.tif]

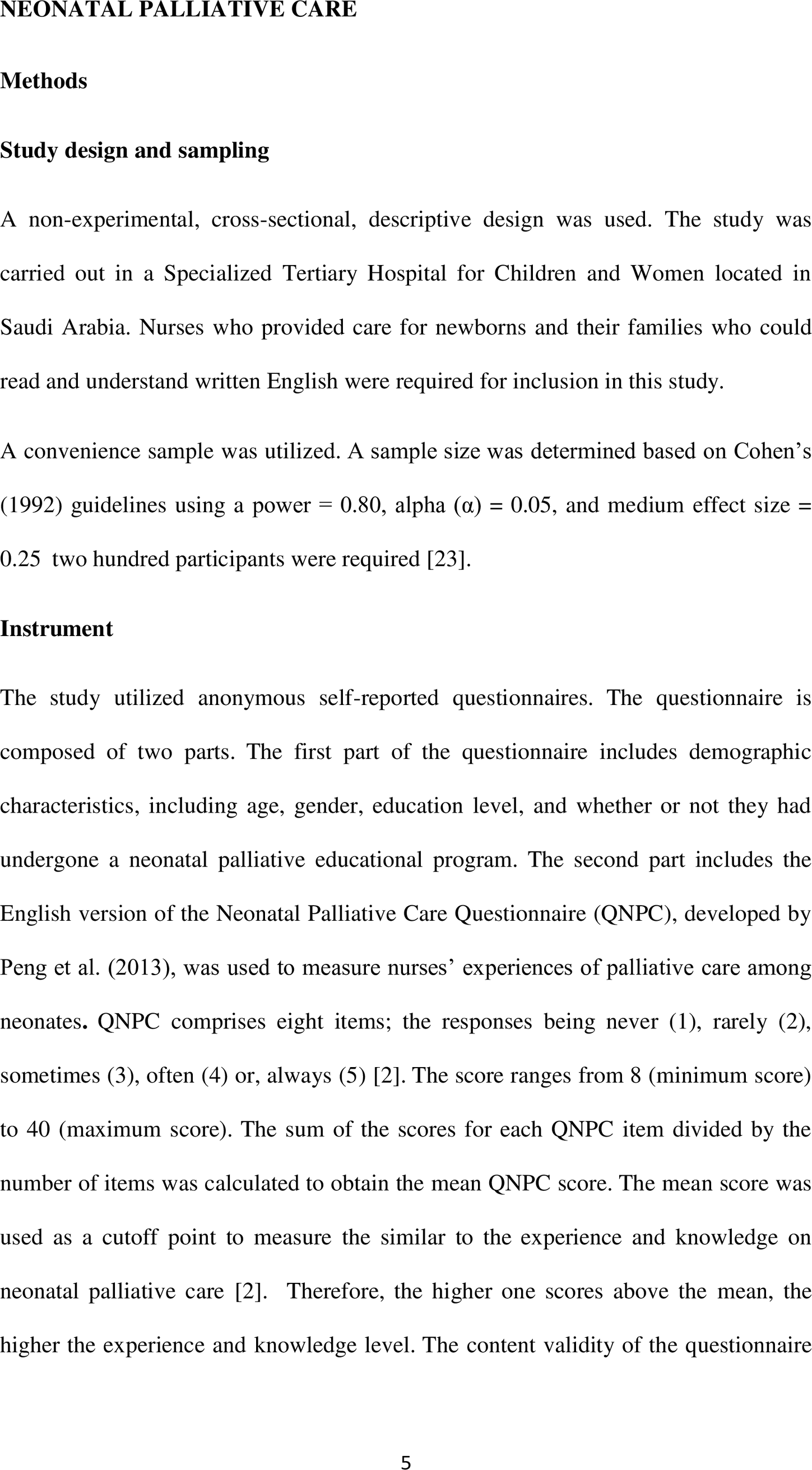

Supplement: S1 File — (ZIP) [file pone.0280081.s002.zip › PACE Corrected/locked tracked changes.tif]

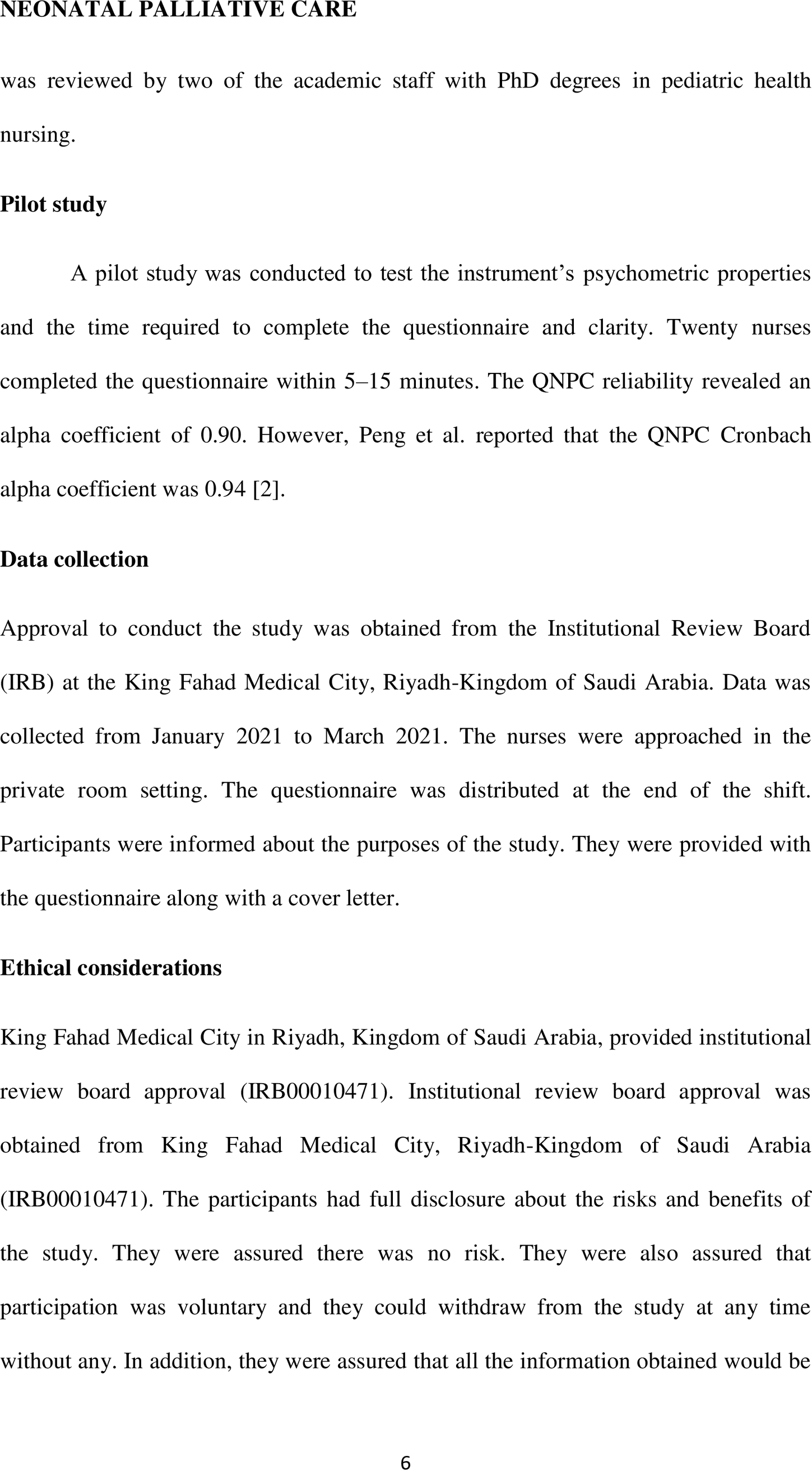

Supplement: S1 File — (ZIP) [file pone.0280081.s002.zip › PACE Corrected/locked tracked changes.tif]

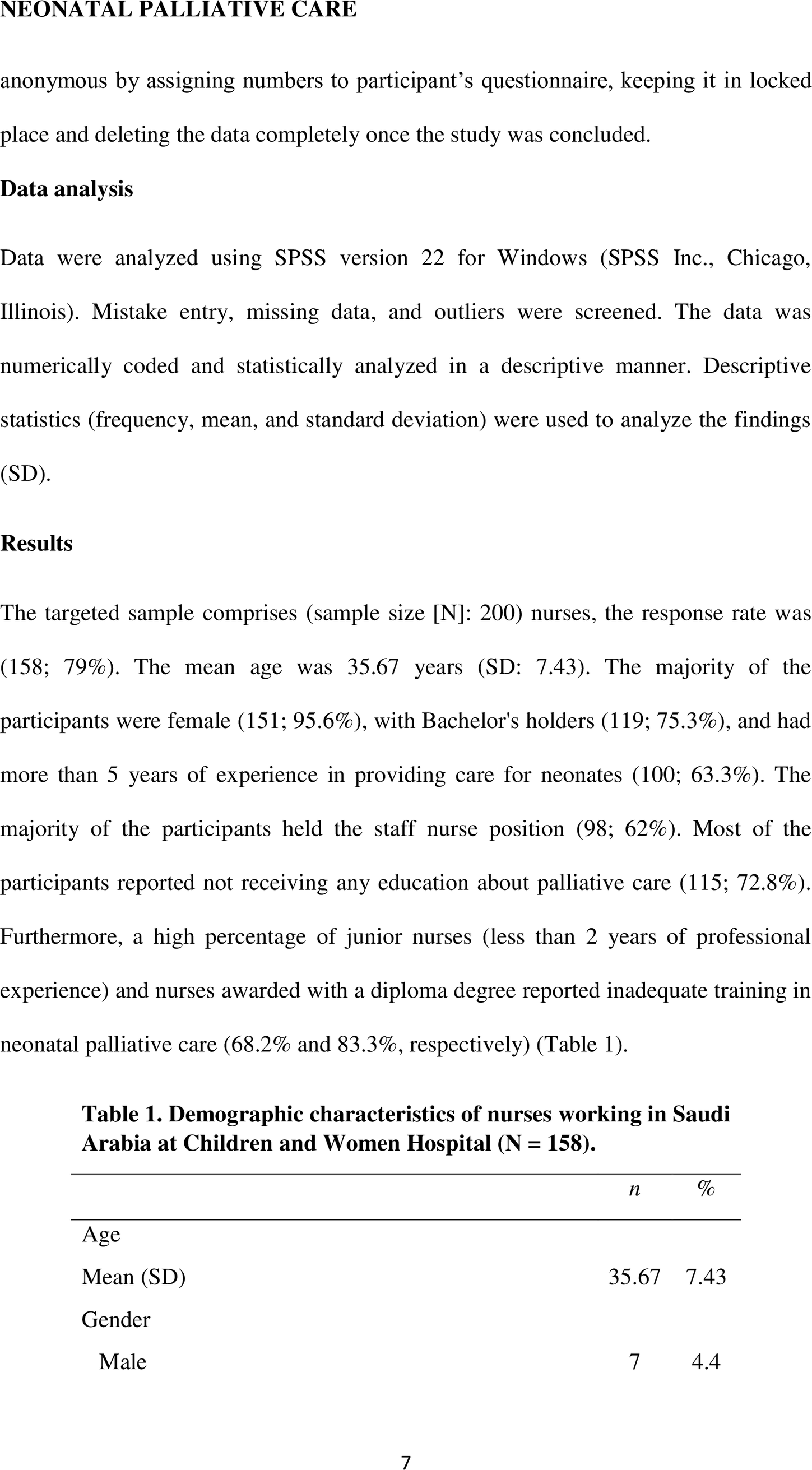

Supplement: S1 File — (ZIP) [file pone.0280081.s002.zip › PACE Corrected/locked tracked changes.tif]

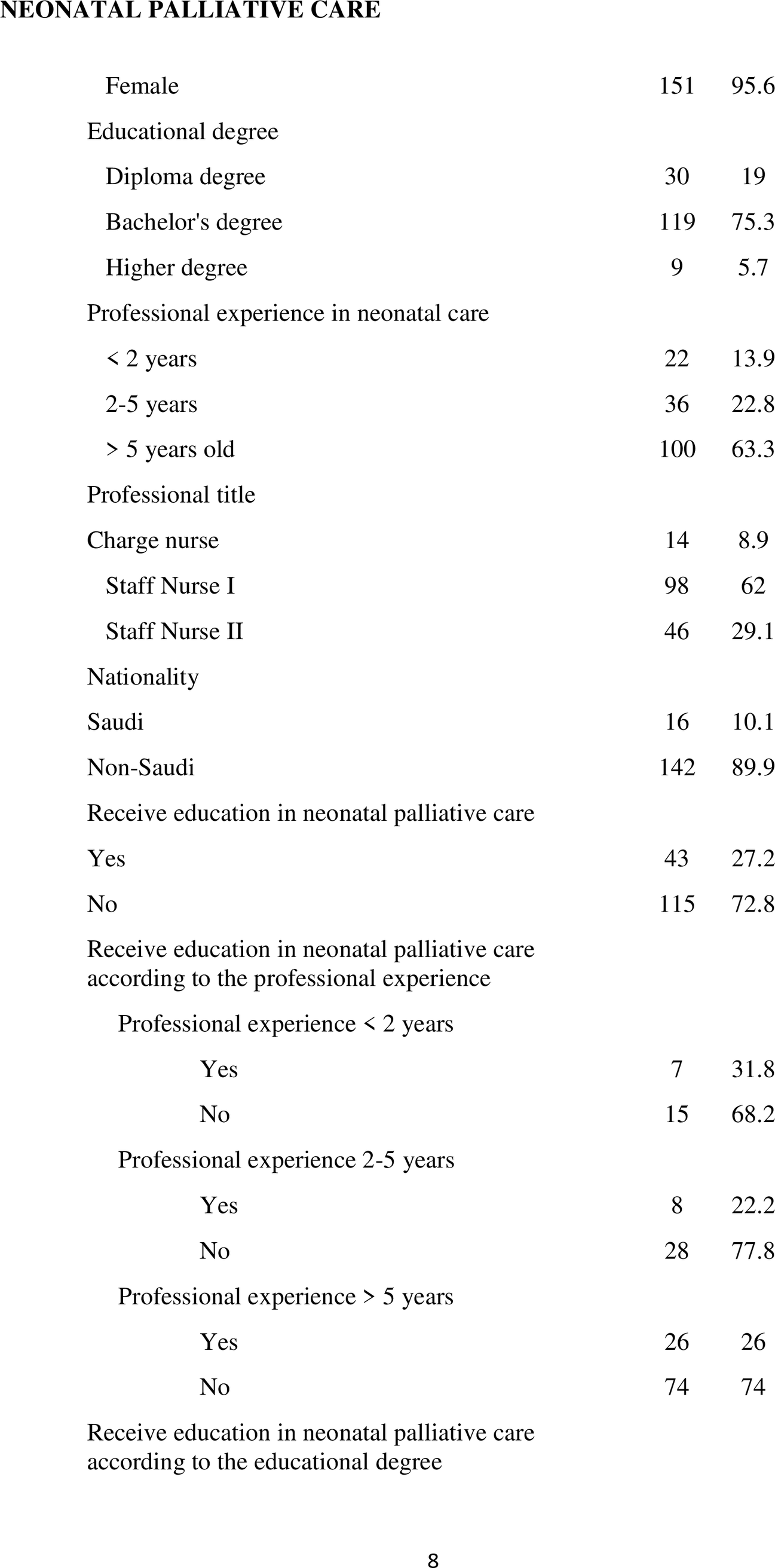

Supplement: S1 File — (ZIP) [file pone.0280081.s002.zip › PACE Corrected/locked tracked changes.tif]

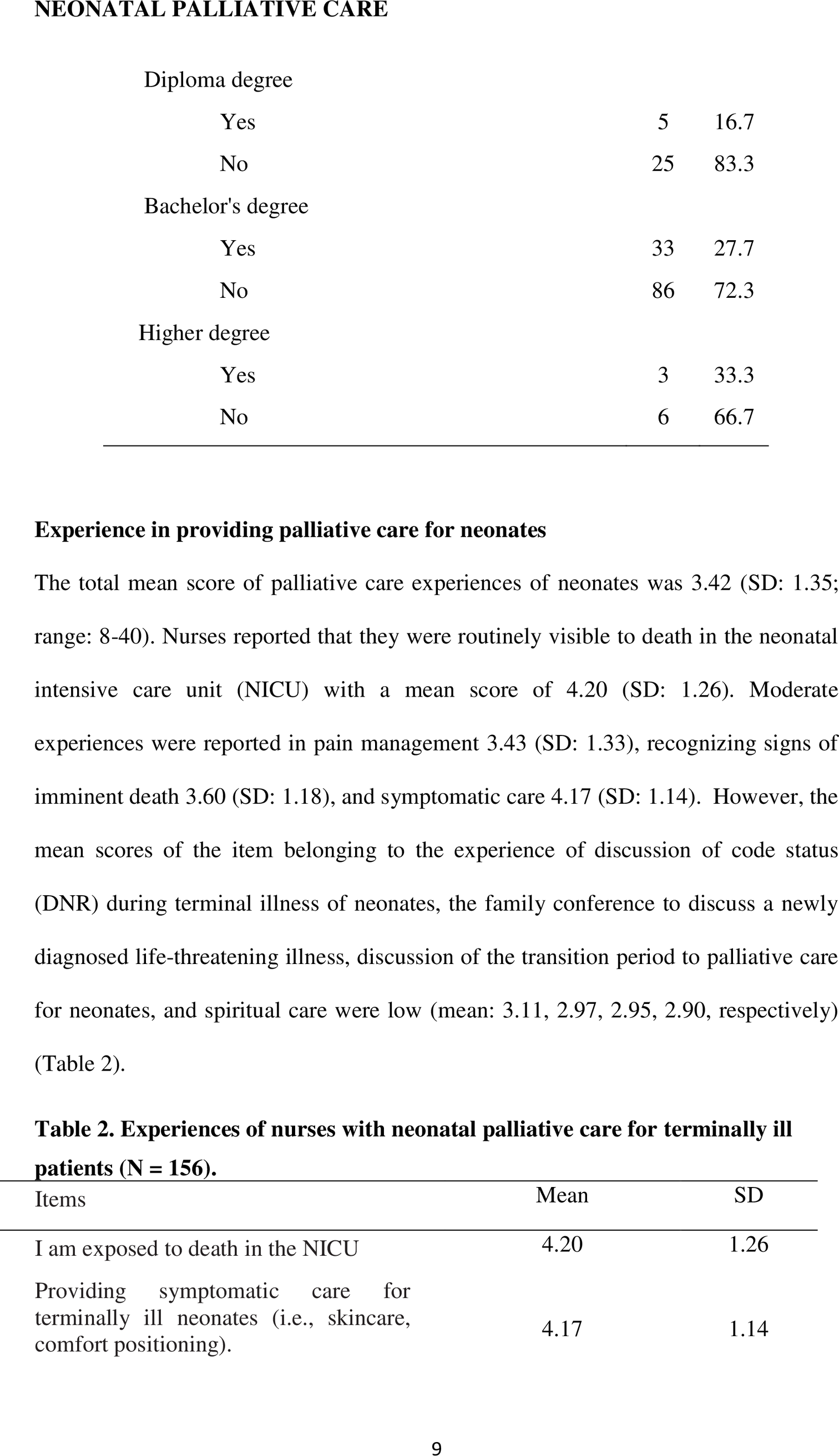

Supplement: S1 File — (ZIP) [file pone.0280081.s002.zip › PACE Corrected/locked tracked changes.tif]

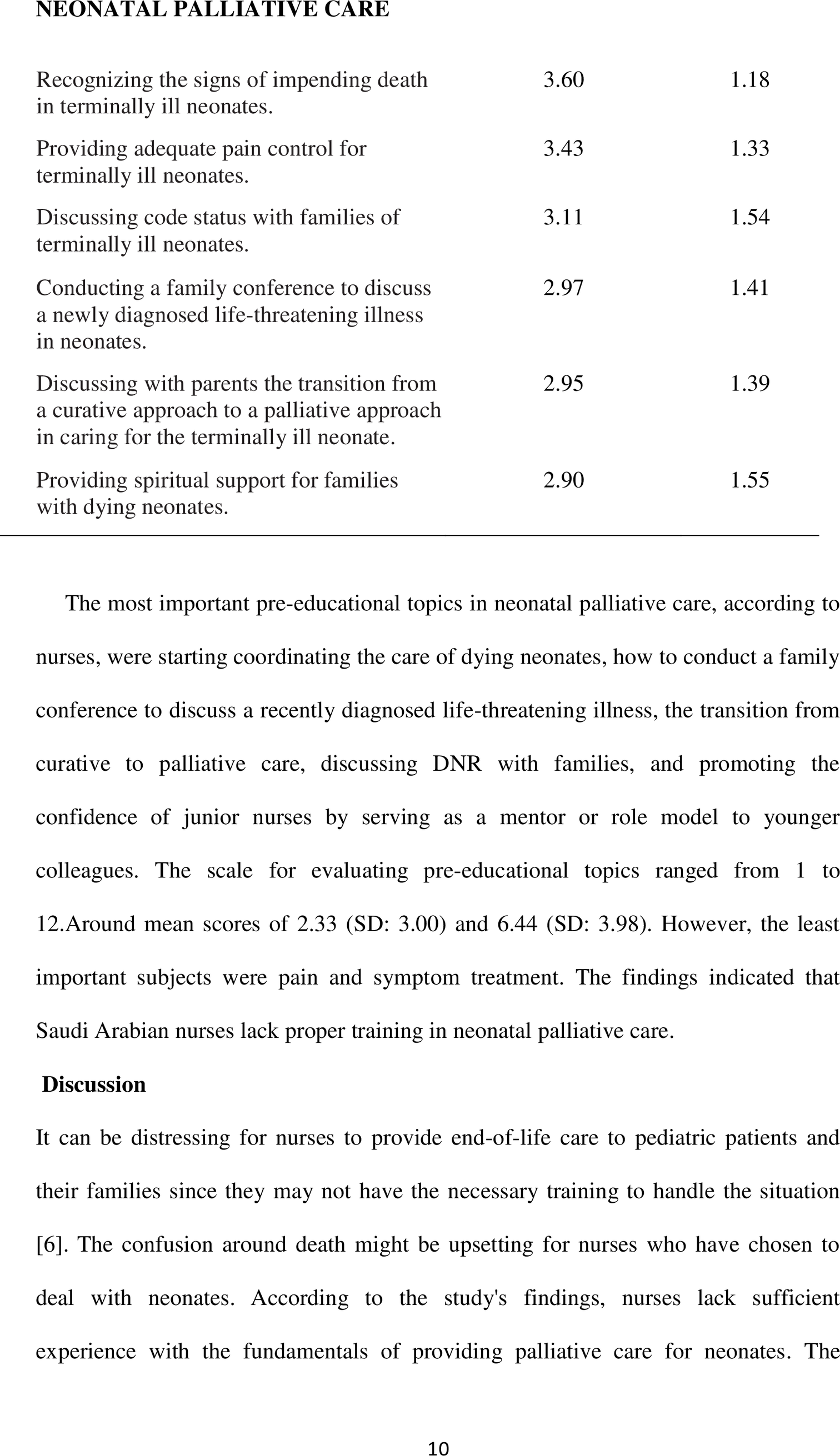

Supplement: S1 File — (ZIP) [file pone.0280081.s002.zip › PACE Corrected/locked tracked changes.tif]

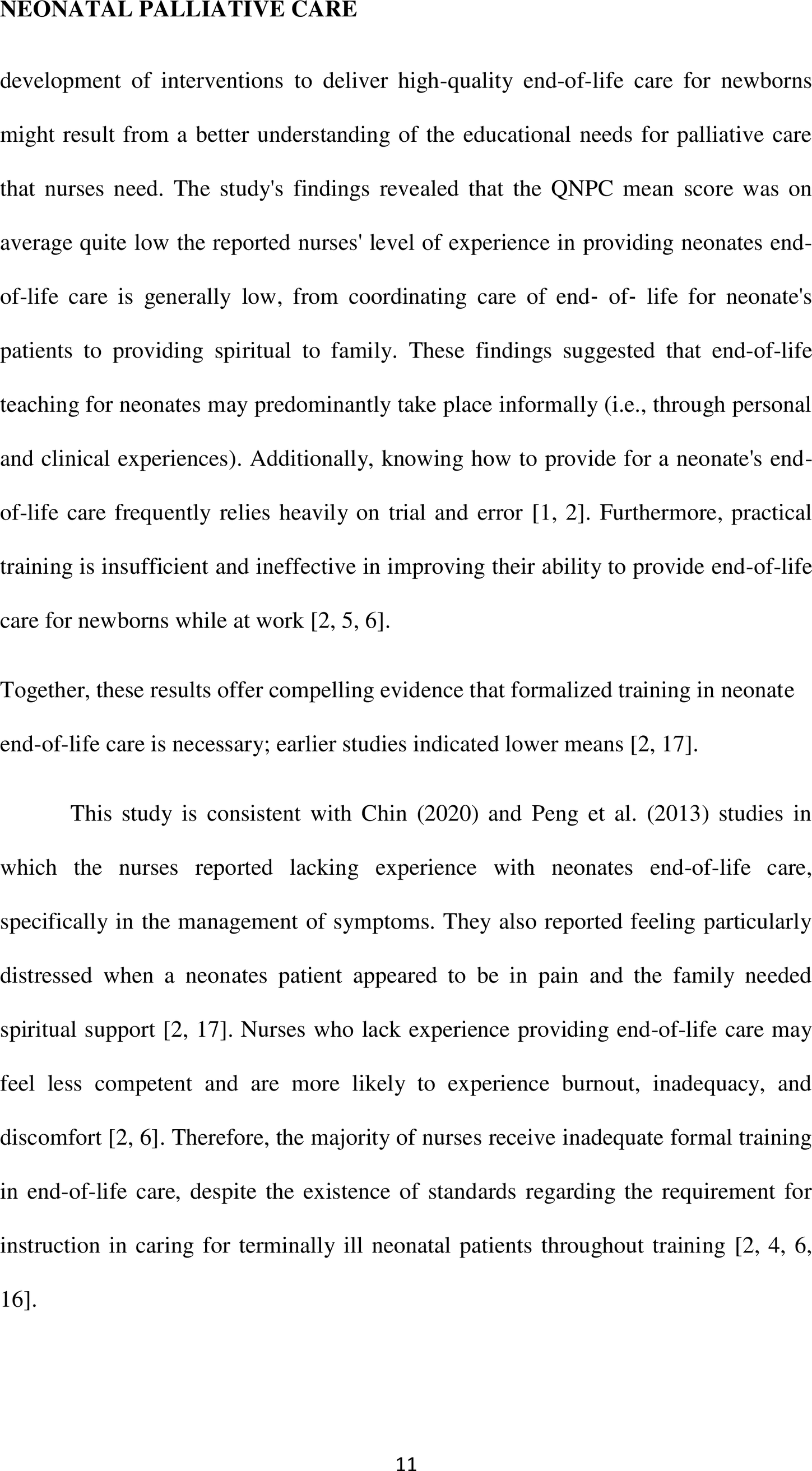

Supplement: S1 File — (ZIP) [file pone.0280081.s002.zip › PACE Corrected/locked tracked changes.tif]

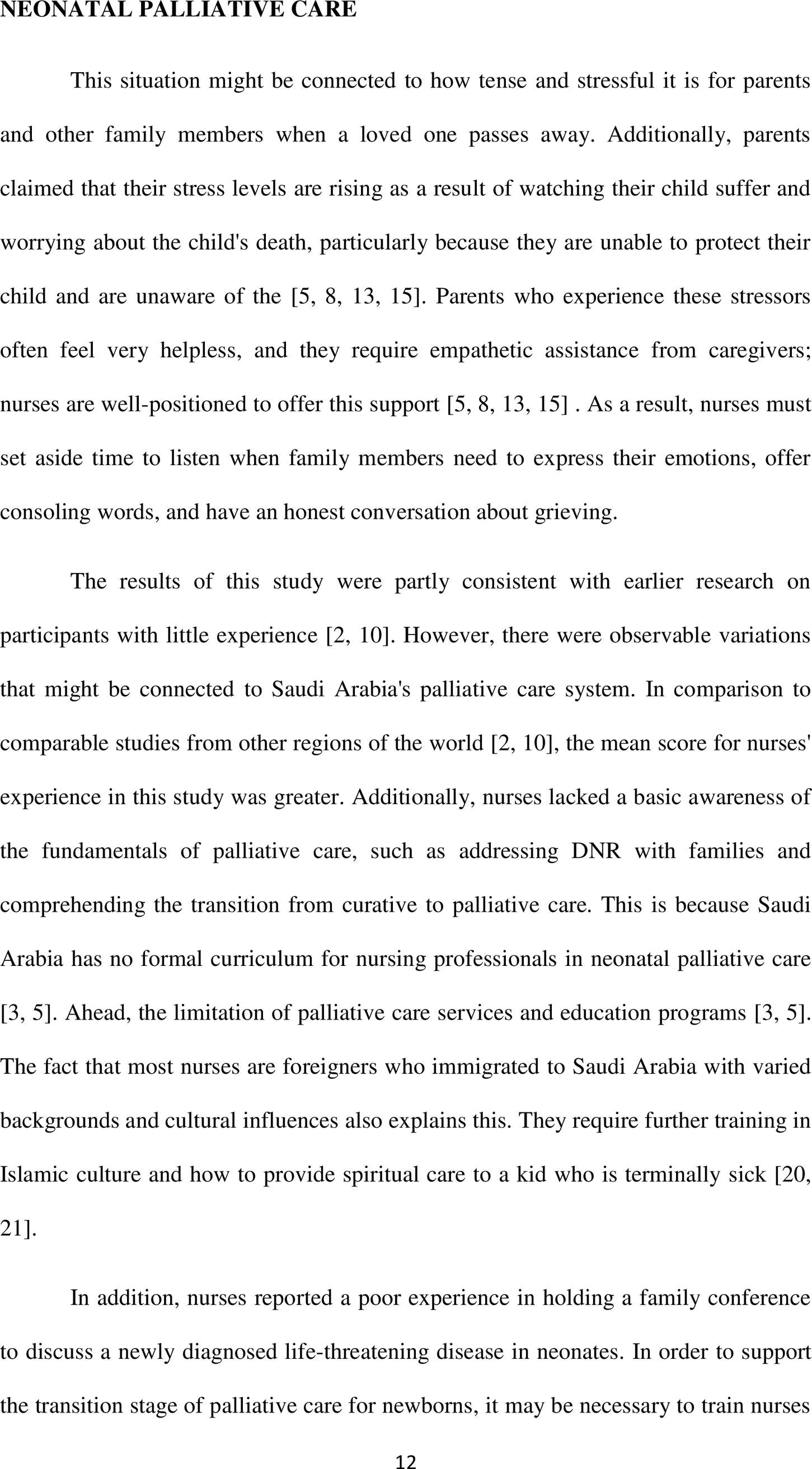

Supplement: S1 File — (ZIP) [file pone.0280081.s002.zip › PACE Corrected/locked tracked changes.tif]

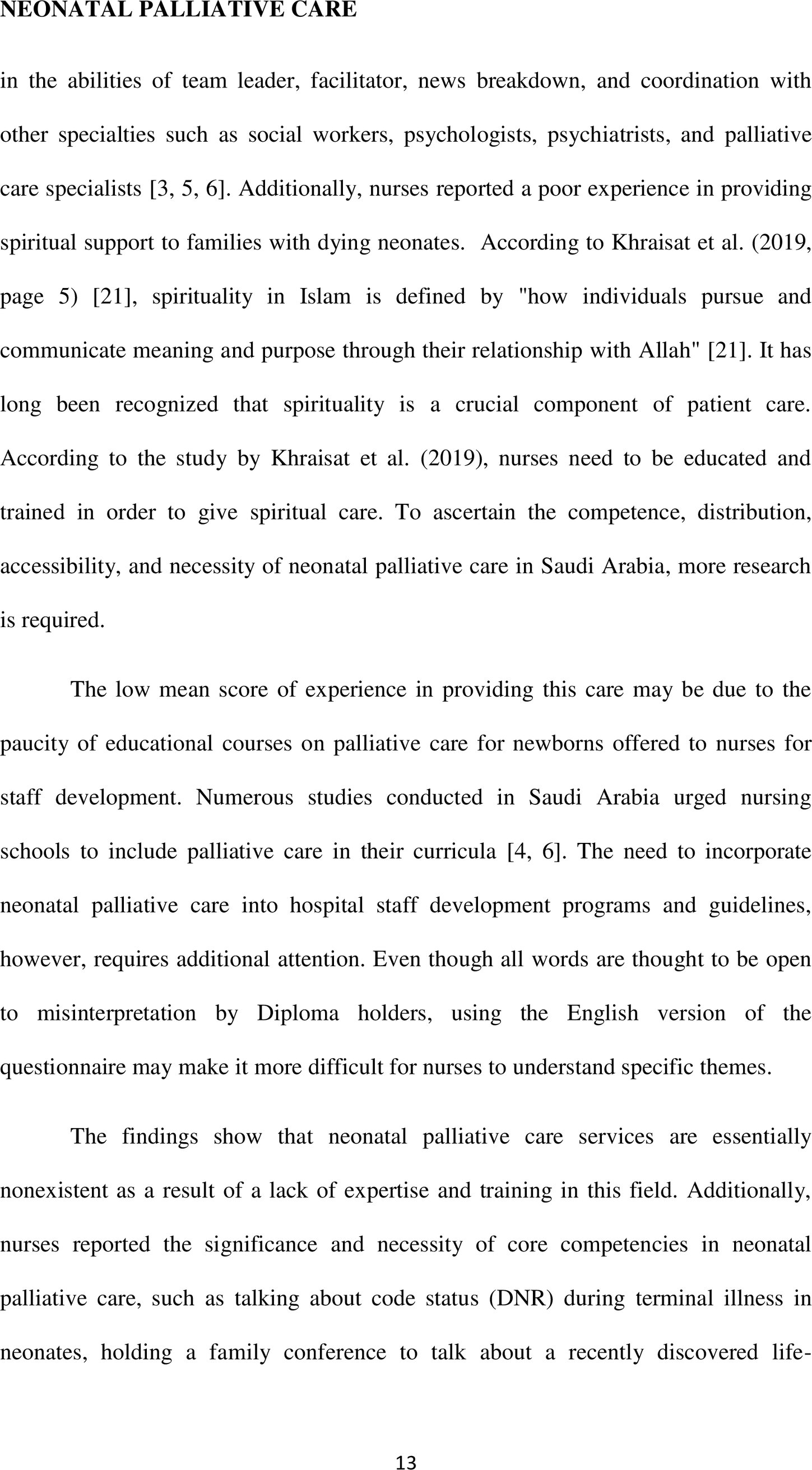

Supplement: S1 File — (ZIP) [file pone.0280081.s002.zip › PACE Corrected/locked tracked changes.tif]

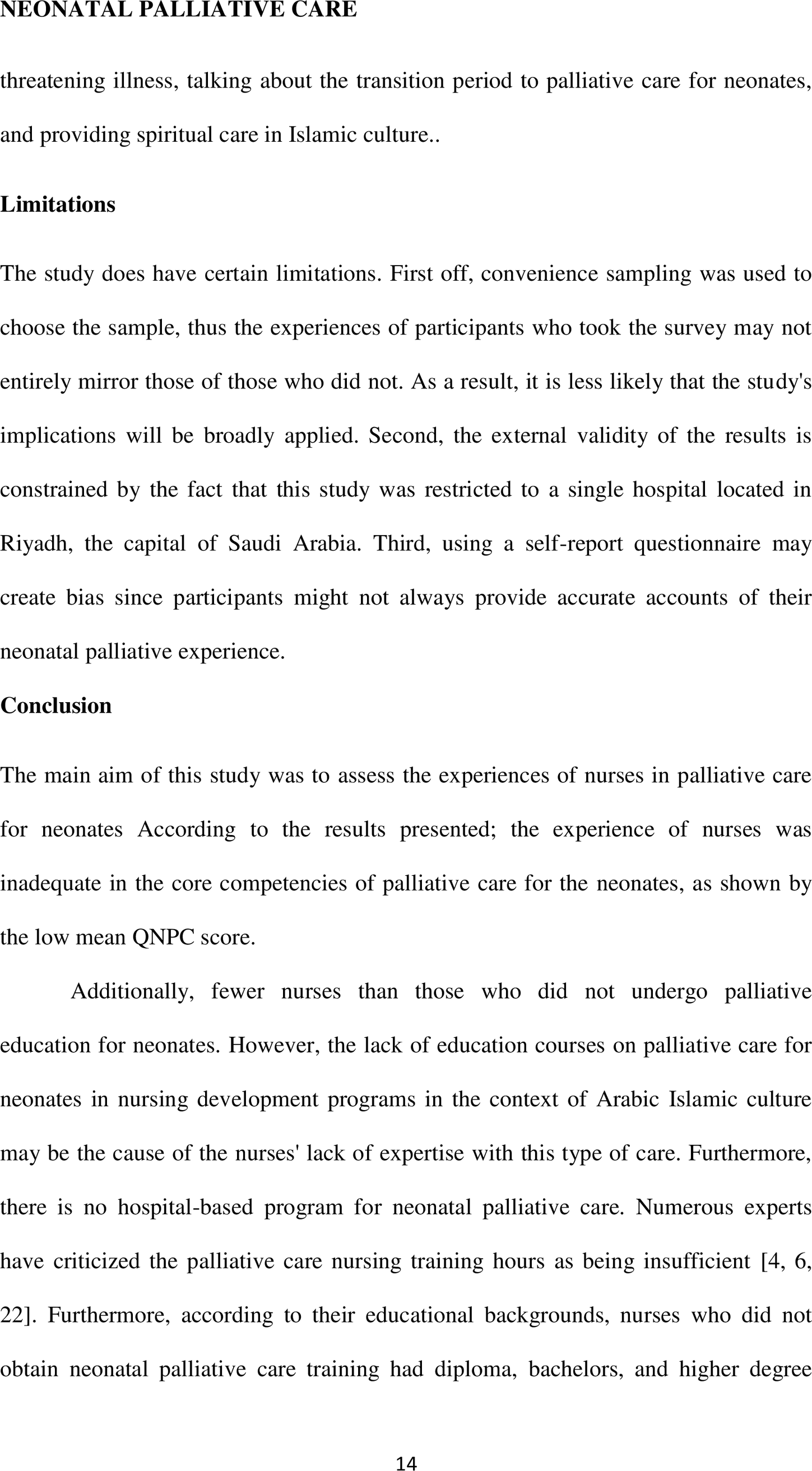

Supplement: S1 File — (ZIP) [file pone.0280081.s002.zip › PACE Corrected/locked tracked changes.tif]

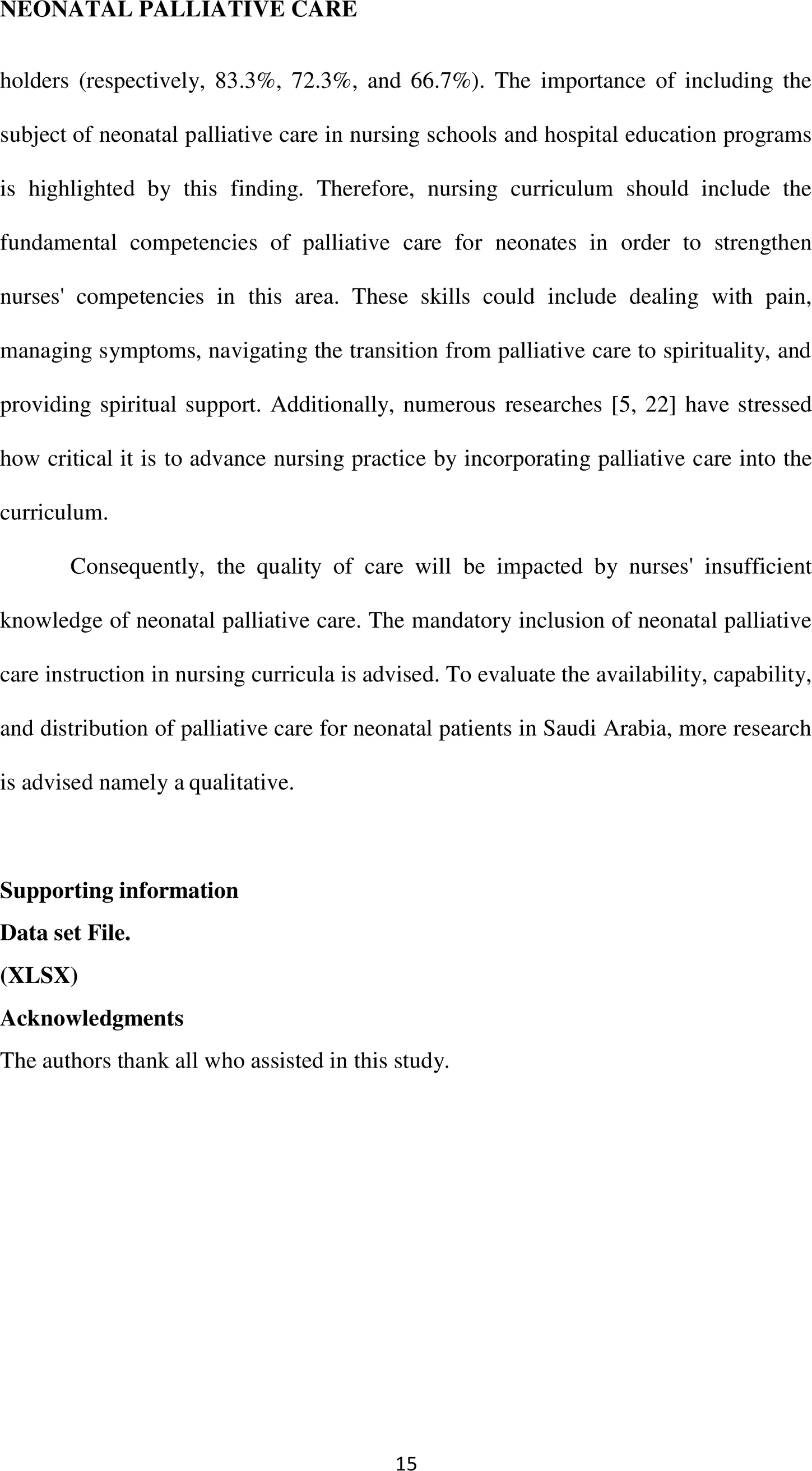

Supplement: S1 File — (ZIP) [file pone.0280081.s002.zip › PACE Corrected/locked tracked changes.tif]

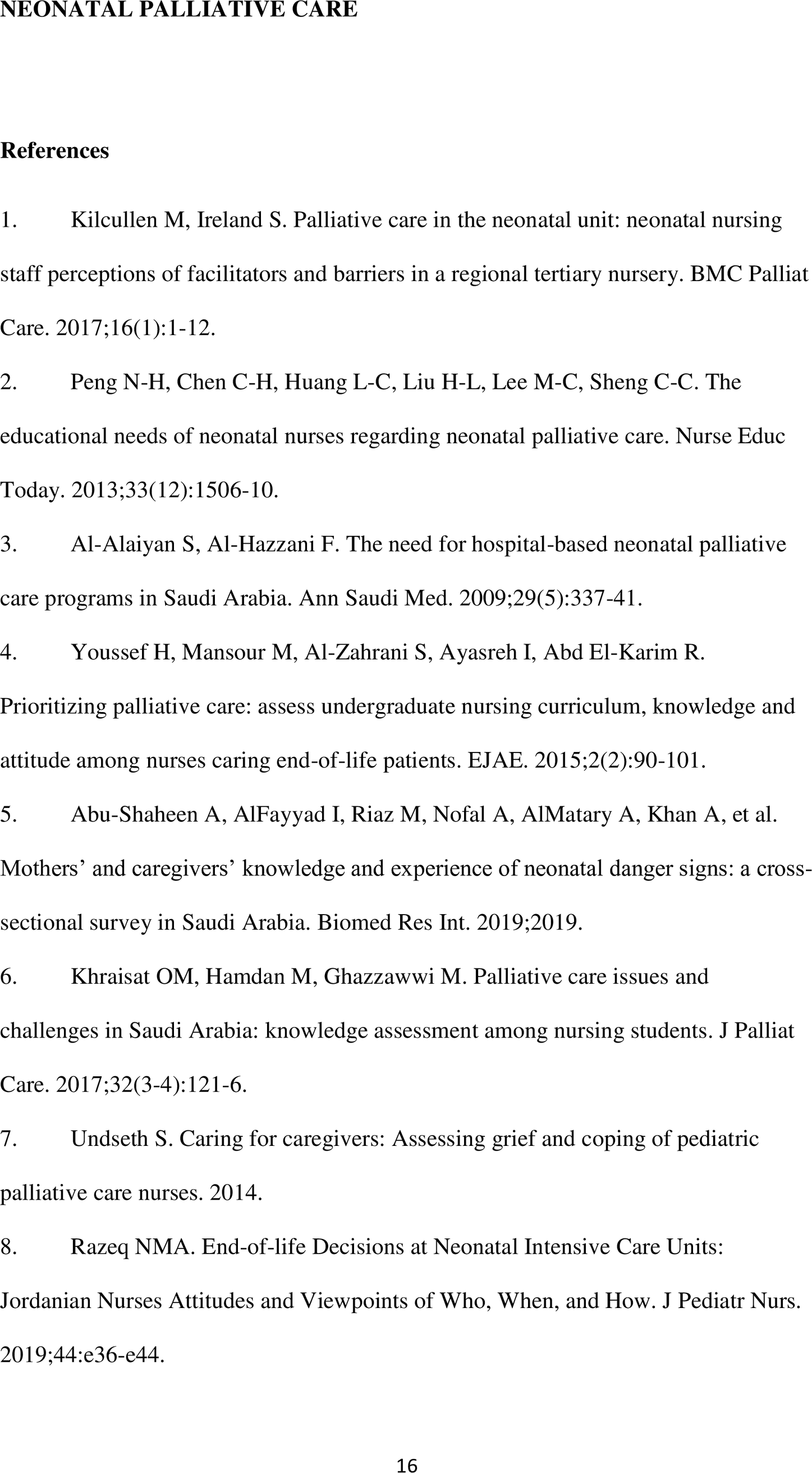

Supplement: S1 File — (ZIP) [file pone.0280081.s002.zip › PACE Corrected/locked tracked changes.tif]

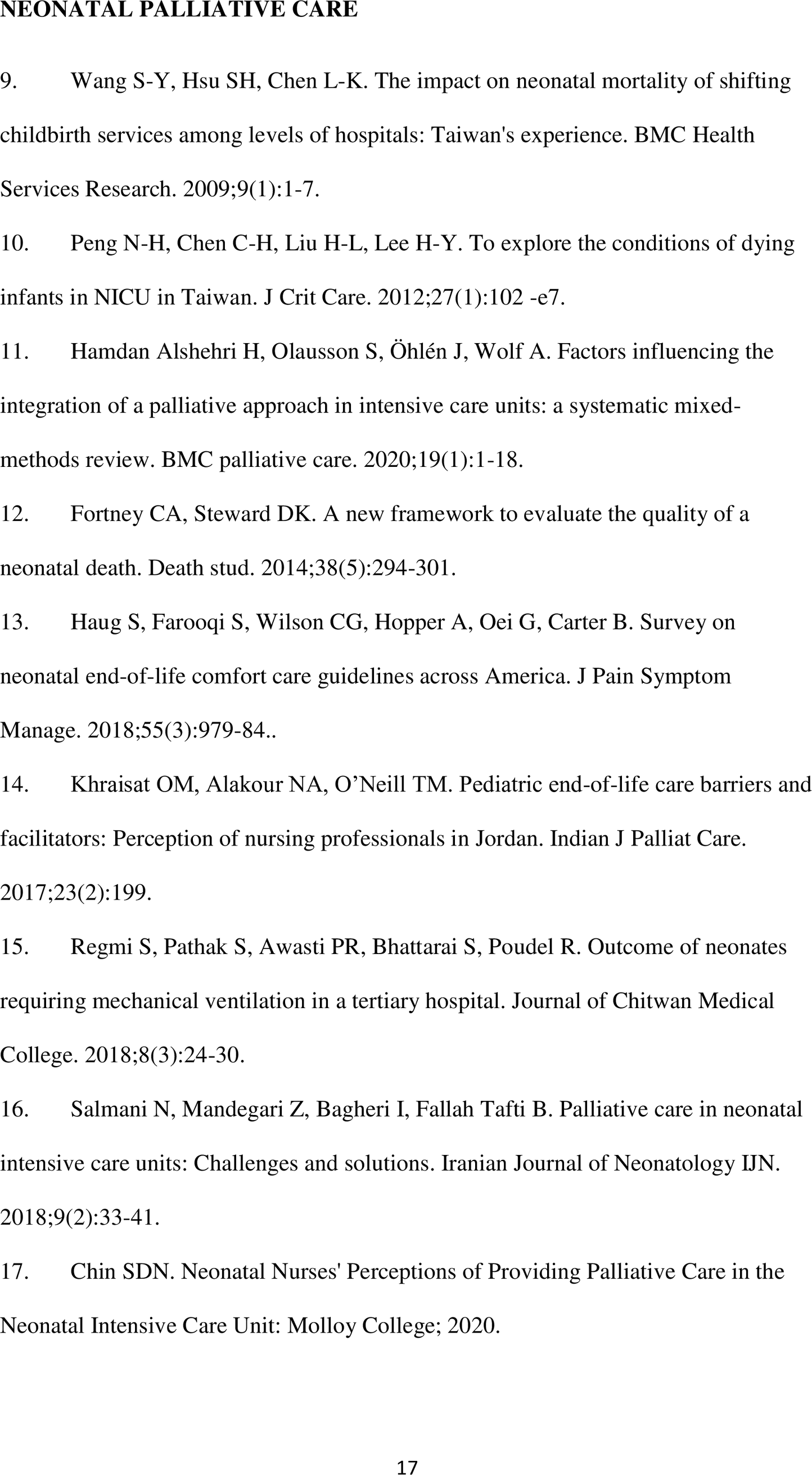

Supplement: S1 File — (ZIP) [file pone.0280081.s002.zip › PACE Corrected/locked tracked changes.tif]

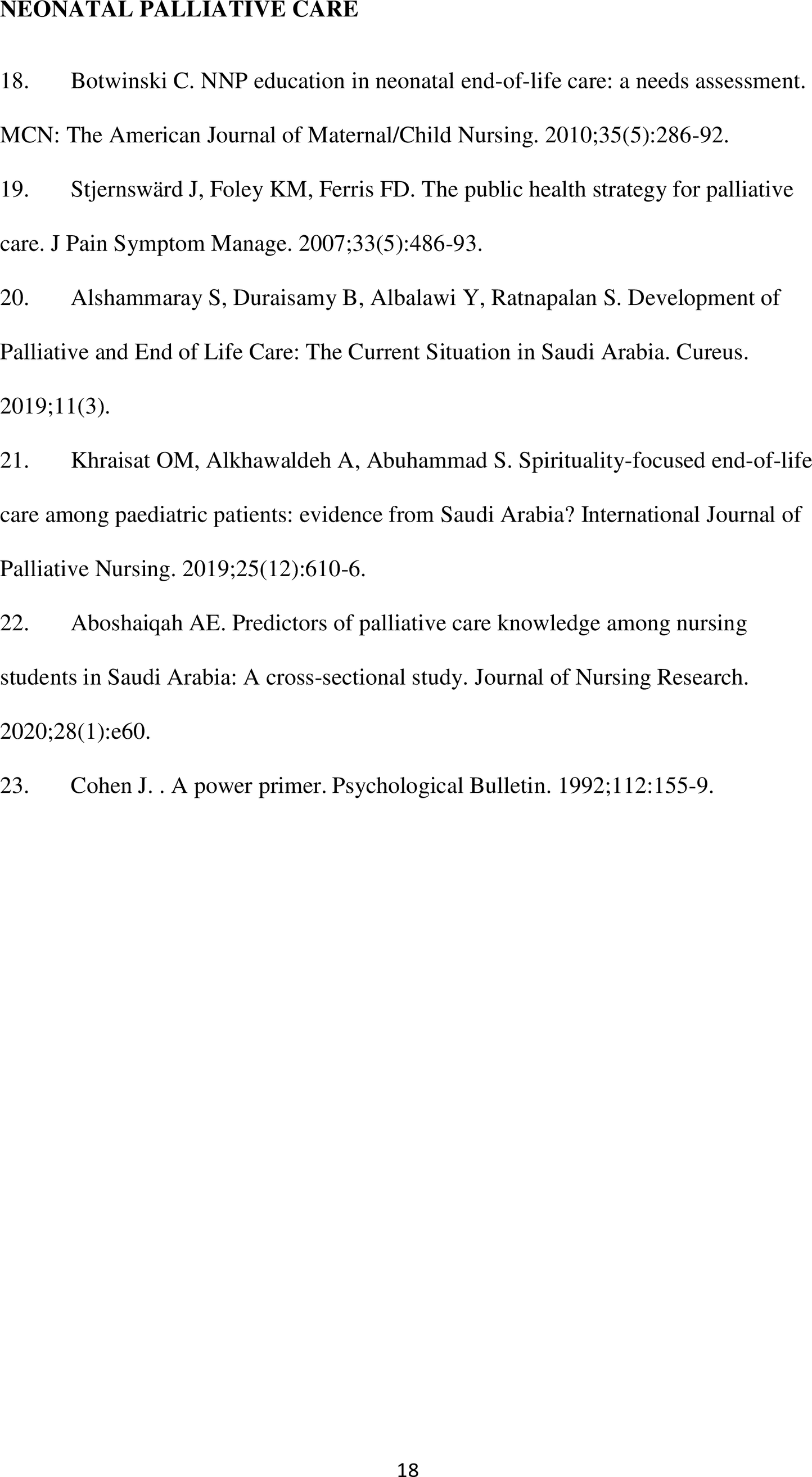

Supplement: S1 File — (ZIP) [file pone.0280081.s002.zip › PACE Corrected/locked tracked changes.tif]
